# Autonomic Dysfunction Following Surgical Resection of Cervicomedullary Hemangioblastoma: A Case Report and Literature Review

**DOI:** 10.7759/cureus.94021

**Published:** 2025-10-07

**Authors:** Naif Alshahrani, Maysoon Alqurashi, Turki Alzidani, Badr E Hafiz, Abdulaziz A Basurrah, Mohammed Aref

**Affiliations:** 1 College of Medicine and Surgery, King Khalid University, Abha, SAU; 2 College of Medicine and Surgery, Umm Al-Qura University, Makkah, SAU; 3 Neurological Surgery, King Faisal Medical Complex, Taif, SAU; 4 Neurosurgery, King Faisal Medical Complex, Taif, SAU; 5 Department of Neurosciences, Section of Neurosurgery, King Faisal Specialist Hospital and Research Centre, Jeddah, SAU; 6 Department of Neurosurgery, King Abdullah Medical City, Makkah, SAU; 7 College of Medicine, Alfaisal University, Riyadh, SAU

**Keywords:** autonomic dysfunction, brainstem surgery, cerebellar hemangioblastoma, cervicomedullary tumor, neurogenic orthostatic hypotension

## Abstract

Hemangioblastomas are rare vascular tumors of the central nervous system, most frequently arising in the cerebellum but occasionally in the spinal cord or brainstem. Their occurrence at the cervicomedullary junction is uncommon yet clinically significant due to the potential for severe neurological and autonomic complications. We report the case of a 63-year-old man who presented with progressive gait ataxia and intermittent loss of consciousness. MRI revealed a 3.3 × 4.5 cm enhancing lesion at the cervicomedullary junction, compressing the brainstem and causing hydrocephalus. Preoperative embolization of feeding vessels from the posterior inferior cerebellar and internal carotid arteries was performed, followed by suboccipital craniotomy and complete tumor excision. Postoperatively, the patient developed recurrent orthostatic hypotension requiring prolonged intensive care, vasopressor therapy, and coordinated multidisciplinary management, consistent with autonomic dysfunction from disruption of brainstem autonomic centers. This case highlights the challenges of managing cervicomedullary hemangioblastomas, emphasizing the need for careful preoperative planning, precise surgical execution, and individualized postoperative care, as even histologically benign tumors can pose life-threatening risks due to their critical location.

## Introduction

Hemangioblastomas are benign, highly vascular, WHO grade 1 tumors that can primarily occur in the central nervous system. Although they are most often found in the cerebellum, they can also occur in the spinal cord or brainstem. These tumors may arise sporadically or as part of von Hippel-Lindau (VHL) disease, an inherited autosomal dominant disorder associated with multiple tumors [1]. Comprising only 1-2.5% of intracranial tumors and 2-10% of spinal cord tumors, they are most commonly diagnosed in individuals aged 30-60 years, with a slight male predominance [2].

The clinical symptoms depend on the tumor's location: cerebellar lesions typically lead to a constellation of signs and symptoms, including ataxia, nystagmus, vertigo, dysarthria, dysmetria, and intentional tremors, in addition to the symptoms of high intracranial pressure as nausea, vomiting, and blurry vision, while involvement of the brainstem can result in motor/sensory deficits, ataxia, or dangerous hemorrhage [2]. Spinal hemangioblastomas generally present with localized pain, weakness, sensory deficits, or dysfunction of the bowel and bladder [3].

Diagnosis is based on MRI, which usually reveals a cystic lesion accompanied by an enhancing mural nodule (60%), but can be purely solid or combined solid and cystic without a mural nodule. Surgical resection is the standard treatment, often preceded by embolization to reduce the risk of bleeding [4]. The prognosis after surgery is typically positive, with recurrence rates below 25%, although patients with VHL need lifelong monitoring due to a higher risk of recurrence [5].

Autonomic dysfunction is a rare but important complication following hemangioblastoma resection, particularly when lesions are located in the brainstem or spinal cord [3]. These tumors may disrupt autonomic centers or pathways during surgery, resulting in labile blood pressure, respiratory irregularities, or impaired thermoregulation. Although often transient, severe cases can significantly affect postoperative recovery and necessitate close perioperative monitoring and supportive management [5].

Here, we present a case that illustrates these challenges and underscore the importance of recognizing and managing autonomic dysfunction following hemangioblastoma resection, along with a literature review of similar reported cases, their management strategies, and outcomes.

## Case presentation

**Figure 1:**
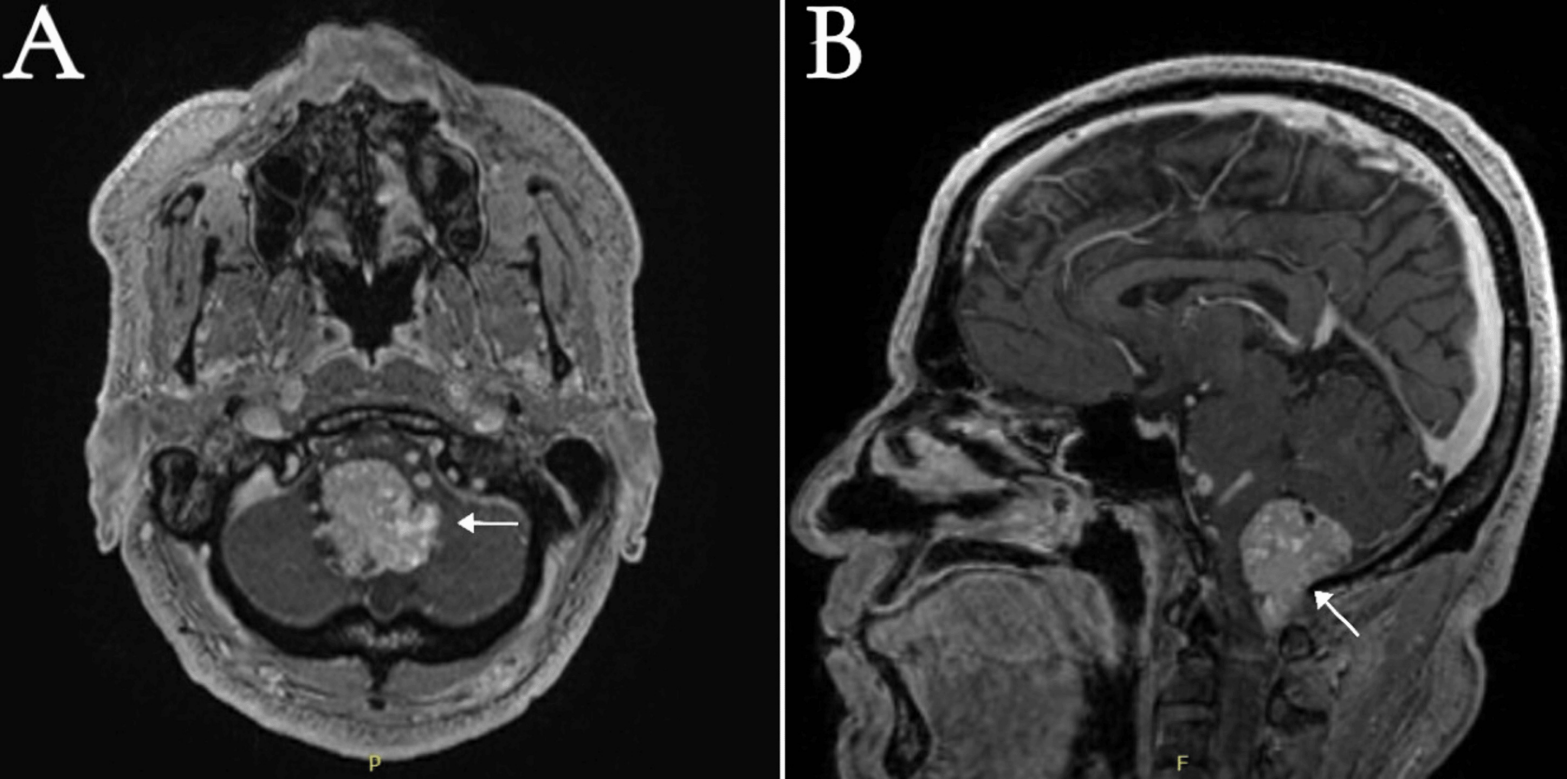
Preoperative post-contrast T1-weighted MRI of the brain: (A) axial and (B) sagittal. The image reveals contrast vividly enhancing an intradural extra-axial solid mass (white arrows) measuring 3.3 × 3.0 × 4.5 cm in the transverse anteroposterior and craniocaudal diameters at the cervicomedullary junction. Multiple large vessels can be noticed within and surrounding the mass in keeping with multiple arterial feeders from the posterior circulation, exerting a significant mass effect on the brainstem and upper cervical cord.

A 63-year-old male was referred to our institution with a three-month history of progressively worsening gait and imbalance, accompanied by episodes of transient loss of consciousness. The patient had previously presented to another hospital, where brain imaging revealed supratentorial hydrocephalus. The initial management included placement of a ventriculoperitoneal (VP) shunt to address elevated intracranial pressure from the hydrocephalus. Upon presentation at our hospital, neurological examination revealed mild truncal ataxia without appendicular involvement, no involvement of the cranial nerve, normal motor strength, sensory perception, and reflex responses. The patient was admitted, and a diagnostic MRI of the brain revealed an intensely enhancing intradural extra-axial mass at the cervicomedullary junction (Figure 1).

**Figure 2:**
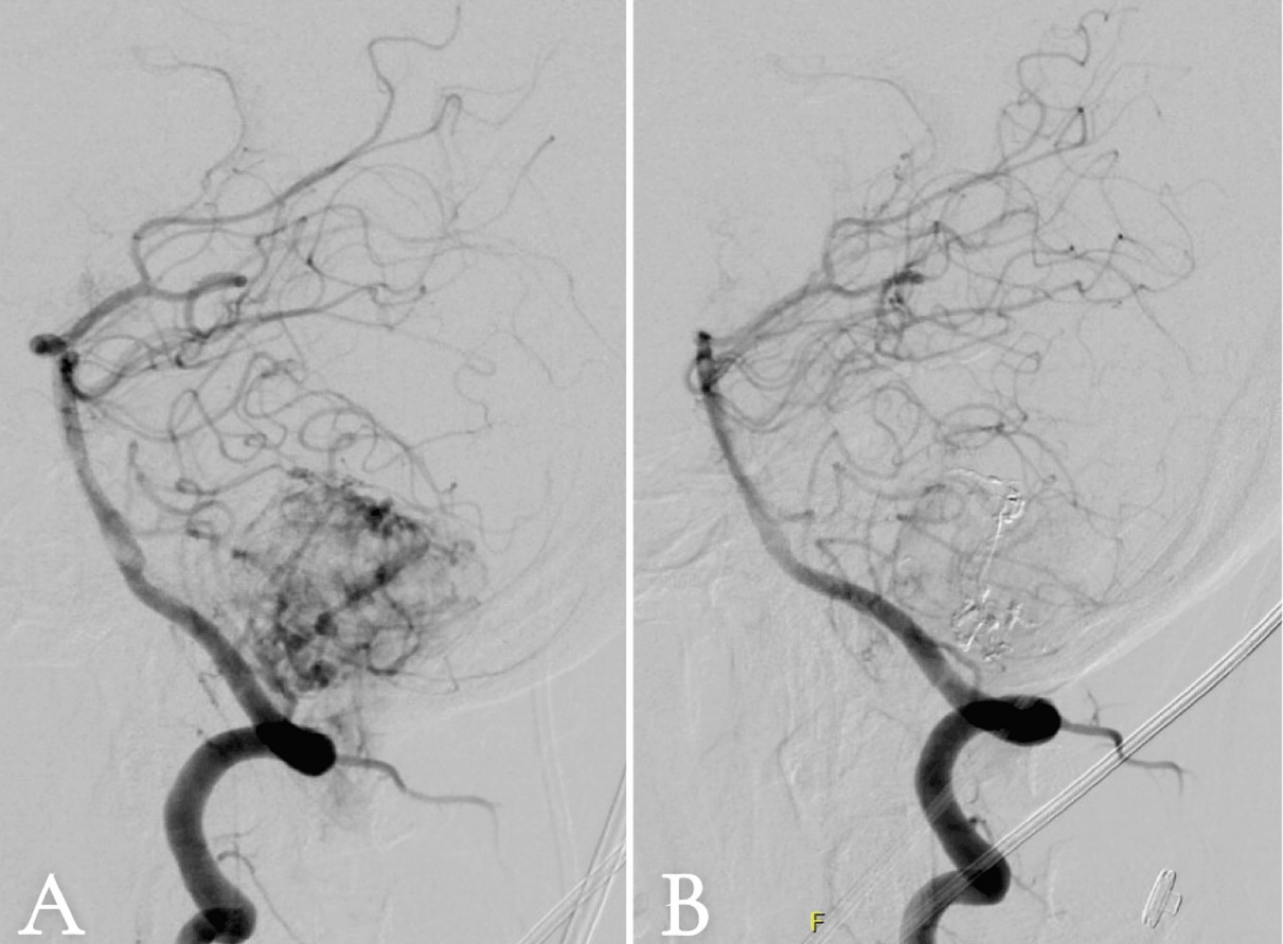
Conventional cerebral angiogram: (A) pre-embolization and (B) post-embolization. Right vertebral artery injection in the lateral view: (A) pre-embolization revealing prominent multiple arterial feeders mainly from the posterior inferior cerebellar artery as well as basilar artery perforators. (B) Post-embolization cerebral angiogram of the right vertebral artery injection in the lateral view revealing embolic material (Onyx) with marked occlusion of the previously described arterial feeders.

Subsequent diagnostic angiography revealed tumor vascular supply from the posterior inferior cerebellar artery (PICA), prompting successful preoperative embolization of these feeding vessels to reduce potential intraoperative hemorrhage (Figure 2).

The patient underwent definitive surgical treatment via a suboccipital craniotomy using a telovelar approach with complete microsurgical resection of the lesion. During the procedure, the previously placed VP shunt was removed and replaced with an external ventricular drain to facilitate controlled cerebrospinal fluid management. The dura was opened with a Y-shaped incision without removing C1. The cerebellar tonsils were carefully mobilized from the foramen magnum, and both PICAs were identified, exposed, and protected with neurosurgical patties. Medullary velum was exposed, and the tumor was peeping and visible immediately after dural opening and was going up to the upper border of C2. The tumor was removed en bloc with identification of the margins and coagulation of the capsule with severance of all feeders. The tumor was attached to the roof of the fourth ventricle. The lower pole of the tumor was lifted off the cord and diathermized, and then, systematically, the tumor margins were defined after opening the thick arachnoid adhesions. The upper border of the tumor was similarly defined and diathermized. Circumferential coagulation was continued with microdissection following the plane between the tumor and brainstem, and the tumor was finally separated en bloc from the brainstem and cerebellum with the intervening roof of the fourth ventricle. No intraoperative neuromonitoring was used.

The surgery was uneventful without any intraoperative complications. The patient was shifted in an intubated state to the surgical intensive care unit (ICU) for observation and strict neuromonitoring. The immediate postoperative course proved challenging, complicated by significant vocal cord edema leading to aspiration pneumonia that necessitated reintubation and nasogastric tube placement. The patient additionally developed recurrent episodes of profound hypotension (systolic blood pressure of 80-70 mmHg, and diastolic blood pressure of 57-45 mmHg) and oxygen desaturation, requiring ICU admission, vasopressor support with dopamine infusion, and high-dose corticosteroid therapy (dexamethasone 10 mg STAT dose followed by 4 mg every six hours). Following stabilization, persistent orthostatic hypotension emerged as a dominant clinical issue. To ensure that tissue perfusion was not compromised despite low systemic blood pressures, the ICU team monitored serial perfusion markers, including mean arterial pressure (MAP), central venous oxygen saturation (ScvO₂), lactate levels, and urine output. These parameters demonstrated adequate end-organ perfusion even when absolute blood pressure values remained low.

A structured workup was performed by neurology, internal medicine, and endocrinology services to rule out alternative causes of hypotension. Cardiac evaluation, including electrocardiogram and echocardiography, excluded arrhythmia and cardiomyopathy. Endocrine testing (thyroid function, cortisol, and adrenal studies) ruled out adrenal insufficiency and thyroid dysfunction. Infectious and metabolic causes were excluded by normal inflammatory markers, electrolytes, and sepsis workup. This was attributed to autonomic dysfunction secondary to manipulation of the cervicomedullary junction during tumor resection, where critical autonomic centers are located.

**Figure 3:**
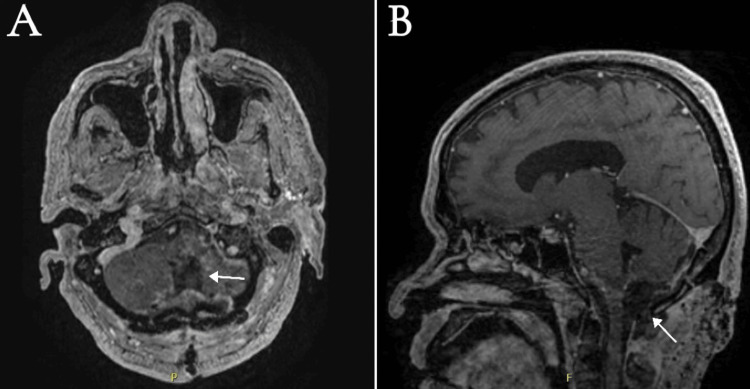
Postoperative postcontrast T1-weighted MRI of the brain: (A) axial and (B) sagittal. The image reveals complete resection of the cervicomedullary junction hemangioblastoma. Posterior fossa postoperative changes with surgical bed mild enhancement and a focal small hemorrhage within the upper part of the medulla (white arrows) can be noted.

Follow-up neuroimaging confirmed complete tumor resection without evidence of residual lesion, hematoma, or recurrent hydrocephalus (Figure 3). The external ventricular drain was removed as the patient was tolerating gradual weaning, and the follow-up image confirmed the absence of hydrocephalus.

Neurological examination postoperatively revealed transient lower limb paresis with 4 out of 5 in the Medical Research Council Scale for Muscle Strength that improved within one week with physical therapy, and the patient’s truncal and appendicular ataxia improved. Over the ensuing two months, a carefully monitored rehabilitation program was implemented. The patient’s vasopressor requirements were gradually tapered while physical therapy was initiated, though initial progress was hindered by recurrent hypotensive episodes. Ultimately, the patient demonstrated meaningful neurological recovery, achieving independent standing and taking steps with assistance. The patient was discharged in good and stable condition. He will be scheduled for an extensive rehabilitation with physical therapy and will be followed in the outpatient clinics regularly to monitor and follow his progression and improvement.

## Discussion

Hemangioblastomas located at the cervicomedullary junction present considerable surgical difficulties due to their closeness to essential brainstem structures and the intricate vascular architecture. Although surgical removal is the most definitive treatment modality, postoperative autonomic dysfunction, while uncommon, can result in serious complications [6]. In our patient, the tumor’s location at the cervicomedullary junction placed autonomic regulatory centers (including the nucleus tractus solitarius and ventrolateral medulla) at risk during resection. Direct surgical manipulation of these areas likely contributed to the development of postoperative hypotension and orthostatic instability.

As demonstrated in our patient, persistent orthostatic hypotension developed after resection of the cervicomedullary hemangioblastoma, complicated by aspiration pneumonia, and necessitated prolonged ICU care, vasopressor support, and intensive rehabilitation. The close proximity of the tumor to autonomic centers within the medulla strongly implicates surgical manipulation in the pathogenesis of this postoperative autonomic dysfunction. While postoperative autonomic dysfunction often presents with significant hypotension, we measured perfusion markers (ScvO₂, lactate, urine output, MAP) in addition to systemic blood pressure, which confirmed that tissue oxygen delivery was preserved despite numerical hypotension. This reinforces the importance of individualized monitoring rather than relying solely on blood pressure thresholds. This highlights the complex interplay between autonomic regulation and hemodynamic stability, reinforcing the need for tailored management strategies that prioritize functional perfusion rather than targeting specific blood pressure readings alone. The patient's eventual improvement despite significant postoperative autonomic instability underscores the value of persistent rehabilitation efforts in such cases. Thus, the cervicomedullary location of the hemangioblastoma was directly linked to the patient’s postoperative autonomic dysfunction, making careful intraoperative handling of brainstem structures and vigilant postoperative monitoring essential in similar cases.

The pathophysiology of hemangioblastomas is characterized by the abnormal growth of vascular endothelial and stromal cells. These cells form distinct nodules and cysts within the spinal cord, which exert mass effects on surrounding neural structures. This compression can lead to clinical manifestations such as localized pain, sensory disturbances, and motor weakness, all of which can profoundly impact a patient’s quality of life [6]. Surgical resection remains the gold-standard treatment for hemangioblastomas, with complete resection being the primary objective to achieve long-term tumor control while minimizing neurological deficits [7]. However, even with modern microsurgical techniques and intraoperative neuromonitoring, approximately 40% of patients suffer transient postoperative neurological worsening, likely due to the tumors’ highly vascular nature and their intimate association with critical neural structures [8].

Orthostatic hypotension is characterized by a decrease in systolic blood pressure of at least 20 mmHg or a drop in diastolic blood pressure of at least 10 mmHg within three minutes of standing up. Its symptoms are often nonspecific and may include dizziness, fainting, pain in the neck and shoulders, and weakness or buckling of the legs [9]. The orthostatic hypotension observed in this patient likely reflects disruption of the baroreceptor reflex arc, a critical regulator of blood pressure homeostasis. This arc comprises afferent pathways (via the glossopharyngeal and vagus nerves), central processing (nucleus tractus solitarius and ventrolateral medulla), and efferent pathways (sympathetic fibers via the intermediolateral cell column and parasympathetic vagal output) [10].

A targeted literature review was conducted using PubMed and indexed neurosurgical journals. Search terms included “hemangioblastoma,” “cranial,” “posterior fossa,” “cervicomedullary,” “autonomic dysfunction,” “orthostatic hypotension,” and “baroreflex failure.” Eligible studies included peer-reviewed case reports, case series, and case lessons describing autonomic dysfunction following the surgical resection of cranial hemangioblastomas. Table 1 presents a literature review of cases that developed autonomic dysfunction following the resection of cranial hemangioblastomas [11-16].

**Table 1:** Literature review of autonomic dysfunction after cranial hemangioblastoma resection. Y: years; M: male; F: female; NR: not reported; CMJ: cervicomedullary junction; HTN: hypertension; OH: orthostatic hypotension; RVLM: rostral ventrolateral medulla; VHL: von Hippel–Lindau disease; BP: blood pressure; Meds: medications

Authors (year)	Study design	Patient /Age and sex	Tumor location	Presenting symptoms	Surgical approach	Autonomic dysfunction	Postoperative outcomes	Complications	Medical therapy	Pathophysiology	Mortality	Key takeaways
Finestone and Teasell (1993) [11]	Case report	18 Y - M	Inferior fourth ventricle (area postrema)	NR (gastric ulcer later noted)	Posterior fossa resection	Autonomic dysreflexia (paroxysmal HTN, bradycardia, flushing, headache)	Recurrent dysreflexia episodes	A gastric ulcer triggered episodes	Supportive care; ulcer treatment	Sympathetic outflow disruption post-medullary surgery	No	Lesions near medullary autonomic centers can cause dysreflexia; addressing triggers improves symptoms
Jabary et al. (2007) [12]	Case report	54 Y - F	Posterior fossa	NR	Hemangioblastoma excision	Severe orthostatic hypotension	Improved with medical therapy	NR	Midodrine	Baroreflex dysfunction	No	Postoperative OH can occur; midodrine is beneficial
Hocker et al. (2012) [13]	Case report	34 Y - F	Dorsal medulla, adherent to obex	NR	Posterior fossa craniotomy	Orthostatic hypotension	Recovered, off BP meds by 3 months	NR	BP agents, rehab	Baroreflex failure from dorsal medullary injury	No	Autonomic dysfunction can be transient; supportive therapy is often effective
Ideguchi et al. (2010) [14]	Case report	21 Y -M	Behind the dorsal medulla	NR	Cerebellar hemangioblastoma excision	Continuous hypertension, tachycardia	Persistent hypertension/tachycardia	NR	Antihypertensives (unspecified)	Sympathetic overactivity at the rostral ventrolateral medulla	No	RVLM involvement may cause a sympathetic storm post-surgery
Nangunoori et al. (2016) [15]	Case report	58 Y - F	Cervicomedullary junction (recurrent)	Progressive neurologic decline	Suboccipital resection; reoperation attempt was aborted	Refractory orthostatic hypotension	Symptomatic improvement with therapy	Later sepsis/aspiration pneumonia	Abdominal binder, fludrocortisone, midodrine	Baroreflex failure from CMJ compression	Yes (sepsis, not directly from OH)	OH can persist long-term; multidisciplinary management is critical
Jacobsen et al. (2021) [16]	Case report	56 Y - M	Cervicomedullary junction	NR	Hemangioblastoma resection	Orthostatic hypotension	Improved with therapy	NR	Fluids, compression; pharmacotherapy (NR)	Baroreceptor response disruption	No	CMJ involvement poses a high risk of OH

Several cases of autonomic dysfunction following hemangioblastoma resection have been reported in the literature (Table 1), most often in association with lesions located at the medulla or cervicomedullary junction. Finestone and Teasell [11] described autonomic dysreflexia after resection of a medullary hemangioblastoma, while Jabary et al. [12] reported severe orthostatic hypotension requiring midodrine therapy following surgery for a dorsal medullary lesion. Similarly, Hocker et al. [13] noted persistent orthostatic hypotension after resection of a medullary hemangioblastoma in a patient with VHL disease. On the other hand, Ideguchi et al. [14] reported a case of a 23-year-old woman who developed persistent hypertension and tachycardia due to delayed and aggressive vasospasm following resection of a left para-pontine cerebellar hemangioblastoma.

Our case shares important parallels with these reports, particularly in the presentation of profound postoperative orthostatic hypotension that necessitated prolonged ICU management and vasopressor support. However, unlike the cases reported by Jabary et al. [12] and Hocker et al. [13], our patient also experienced aspiration pneumonia and recurrent desaturation episodes, complicating the clinical course. Nangunoori et al. [15] described refractory hypotension after cervicomedullary hemangioblastoma resection, and Jacobsen et al. [16] similarly reported persistent orthostatic dysfunction following cervicomedullary tumor surgery; both are more directly comparable to our case, given the tumor location at the cervicomedullary junction.

The unique aspect of our case lies in the detailed hemodynamic monitoring and systematic exclusion of alternative causes of hypotension, which provided evidence that autonomic dysfunction due to brainstem manipulation was the primary etiology. Furthermore, our patient demonstrated gradual neurological and functional recovery with an extended rehabilitation program, which emphasizes the potential for favorable outcomes even after severe postoperative autonomic instability.

While previous reports confirm that autonomic dysfunction is a recognized but rare complication of hemangioblastoma surgery, our case adds to the literature by underscoring the critical role of tumor location at the cervicomedullary junction, the importance of multimodal perfusion monitoring, and the necessity of aggressive supportive care and rehabilitation.

## Conclusions

This case highlights the complex interplay between meticulous preoperative planning, surgical technique, neuroanatomy, and postoperative physiology in the management of cervicomedullary hemangioblastomas. Despite the benign histology of these tumors, their location near critical brainstem autonomic centers poses significant risks. It signifies the importance of using neurophysiological monitoring and all measures that allow for maximal and safe resection of tumors in critical areas. It also illustrates the challenges of autonomic instability following cervicomedullary tumor resection and underscores the importance of individualized management. The observation of low systemic blood pressure did not necessarily correlate with inadequate perfusion and highlights the need to assess patients holistically, considering autonomic regulation alongside hemodynamic parameters to guide clinical decision-making effectively. This also emphasizes the need for a high index of suspicion in evaluating such patients alongside the coordinated multidisciplinary team involvement for such a rare and complex phenomenon.
